# Willingness to pay... What???

**DOI:** 10.1590/1516-3180.2014.1323844

**Published:** 2014-04-14

**Authors:** Alessandro Wasum Mariani, Paulo Manuel Pêgo-Fernandes

**Affiliations:** I MD. Thoracic Surgeon, Instituto do Coração (InCor), Hospital das Clínicas (HC), Faculdade de Medicina da Universidade de São Paulo (FMUSP), São Paulo, Brazil.; II MD, PhD. Associate Professor, Discipline of Thoracic Surgery, Instituto do Coração (InCor), Hospital das Clínicas (HC), Faculdade de Medicina da Universidade de São Paulo (FMUSP), São Paulo, Brazil.

Willingness to pay is a term used in economics, which can be defined as the maximum amount a person would be prepared to pay, sacrifice or exchange in order to receive goods or services or to avoid something that is undesired. It can be used in medicine as a method for assessing the value of health benefits in a cost-benefit analysis.

One indication of the importance of this concept is the progressive appearance of this term in the National Institutes of Health’s PubMed database. The first appearance was in 1972, but it then remained uncommon, with less than 10 mentions per year until the 1990s. From 2000 to 2010, the numbers of appearances of this term grew from 69 times a year to 213 times a year. In 2013, this term appeared 355 times in the PubMed database.

The greatest usages of willingness to pay within medicine are in Pharmacoeconomics and Health Economics. Nonetheless, this term can be a valuable addition within any field in which cost-benefit analysis is desired. Examples of this usage in 2013, retrieved from PubMed, include:

- Patients’ willingness to pay for Alzheimer’s disease medication in Canada.^1^
- Parent preference in Switzerland for easy-to-use attributes of growth hormone injection devices quantified according to willingness to pay.^2^
- Willingness to pay for anterior cruciate ligament reconstruction.^3^

Some authors have taken the view that willingness to pay is a valuable tool in performing cost-benefit analysis for evaluating new healthcare interventions. One of the advantages of willingness to pay is its relative simplicity, given that it can be ascertained through a simple survey.^4^

In 2001, Olsen and Smith published a paper on a major review in which 71 willingness-to-pay surveys relating to healthcare that had been published in English between 1985 and 1998 were gathered together. The authors’ aim was to evaluate willingness to pay against another tool (quality-adjusted life-years) as a measurement of the benefit of healthcare programs. According to these authors, the most important argument for using willingness to pay was that it enabled a more comprehensive valuation of benefits than was possible with quality-adjusted life-years.^5^

However, not all authors have agreed regarding the validity and usefulness of willingness to pay. Burrows and Brown^6^ defended the position that willingness-to-pay analysis can produce widely varying values. They emphasized that there are too few studies to validate the technique, and that the methodologies for eliciting values are underdeveloped. In the conclusion of their paper, they stated: “until its validity has been established, willingness to pay must be rejected as a measure of economic value”.^6^

We can conclude that willingness to pay is an interesting concept that may be very helpful in defining the directions of healthcare policies, especially with regard to publicly-funded healthcare. However, like any other research tool, it has limitations. Better understanding of the willingness-to-pay concept, among medical researchers, can be profitable for all of society.
